# An optimized features selection approach based on Manta Ray Foraging Optimization (MRFO) method for parasite malaria classification

**DOI:** 10.3389/fpubh.2022.969268

**Published:** 2022-09-06

**Authors:** Javeria Amin, Muhammad Sharif, Ghulam Ali Mallah, Steven L. Fernandes

**Affiliations:** ^1^Department of Computer Science, University of Wah, Wah Cantt, Pakistan; ^2^Department of Computer Science, COMSATS University Islamabad, Islamabad, Pakistan; ^3^Department of Computer Science, Shah Abdul Latif University, Khairpur, Pakistan; ^4^Department of Computer Science, Design and Journalism, Creighton University, Omaha, NE, United States

**Keywords:** clusters, malaria, K-mean, MRFO, features

## Abstract

Malaria is a serious and lethal disease that has been reported by the World Health Organization (WHO), with an estimated 219 million new cases and 435,000 deaths globally. The most frequent malaria detection method relies mainly on the specialists who examine the samples under a microscope. Therefore, a computerized malaria diagnosis system is required. In this article, malaria cell segmentation and classification methods are proposed. The malaria cells are segmented using a color-based k-mean clustering approach on the selected number of clusters. After segmentation, deep features are extracted using pre-trained models such as efficient-net-b0 and shuffle-net, and the best features are selected using the Manta-Ray Foraging Optimization (MRFO) method. Two experiments are performed for classification using 10-fold cross-validation, the first experiment is based on the best features selected from the pre-trained models individually, while the second experiment is performed based on the selection of best features from the fusion of extracted features using both pre-trained models. The proposed method provided an accuracy of 99.2% for classification using the linear kernel of the SVM classifier. An empirical study demonstrates that the fused features vector results are better as compared to the individual best-selected features vector and the existing latest methods published so far.

## Introduction

Approximately 200 million people die from the mosquito-borne disease malaria every year. It claimed the lives of 405,000 people in 2018, accounting for almost 67% of all children under the age of five (1). It is carried on by the Plasmodium virus, which has four distinct species that can infect people. The disease has been labeled endemic across 38 different parts of the globe. Initial malaria symptoms are fever, chills, vomiting/headache might be potentially moderate and hard to diagnose. The non-treatment of malaria produces sickness and leads to death (2). Malaria is generally linked to poverty, and it is the most common disease in developing countries. The gold standard for malaria diagnosis is microscopic sliding inspection; however, an alternative is a polymerase chain reaction (PCR) test (3). The examination through the sliding microscope method is commonly used for malaria diagnosis. The sensitivity of this method has relied on the pathologist's expertise. The manual slides examination is a tedious and time-consuming task that leads to misdiagnosis. This problem is tackled through a low-cost computer-aided diagnosis system (4). The red blood cells are segmented by a neural network that provides an accuracy of 93.72% (5). The convolutional neural network has been used for malaria detection with an accuracy of 75.39% (6). The deep learning algorithm has been embedded on mobile devices that might be utilized as an application for online malaria cell detection. The proposed technique consists of two steps, where the minimum global screening method is employed for the screening of malaria slides that are subsequently used with CNN with scratch for infected cell classification which provided prediction accuracy of 93.46% (7). The supervised/unsupervised learning models are widely used for the analysis of malaria cells. Several techniques have been developed to detect malaria, but there is still a void in this field, in which larger variation appears among the malaria cells. Furthermore, pertinent feature extraction, as well as the selection, is a challenge for accurate malaria cell segmentation and classification (8). The microscopic images of malaria have noise, poor contrast, illumination, stain, low quality, variations in intensity, size, and irregularity within the region of interest. That's why accurate detection of malaria is a challenging task (9). Therefore, the microscopic malaria images are preprocessed to improve their quality for accurate detection of malaria. After that, hand-crafted features are fused with the deep features and the best features are selected by using the MRFO method, which provides good malaria classification results. Therefore, in this research, a novel model has been proposed for segmentation and malaria cell classification. The foremost contribution steps are described as:

1. Color-based K-mean clustering is applied to three selected clusters to segment the malaria cells
2. Deep extracted features from segmented images are serially fused and then selected active features using MRFO for malaria cell classification.

Article organization is defined as Section Related works discusses the related work; Section Proposed methodology elaborates on the proposed method steps and results are manifested in Section Results and discussion and finally, the conclusion is written in Section Conclusion.

## Related works

In the literature, several optimizations, clustering, and classification techniques are widely used for the analysis of malaria cells, some of which are discussed in this section (8, 10–14). The classification techniques used for the diagnosis of the malaria cells, in which AdaBoost (15), Naïve Bayes Tree (16), SVM (17), DT (18), and Linear Discriminant (19), classifiers are involved. Custom convolutional neural models are widely used for the analysis of malaria cells and provide an accuracy of 97.37%. Furthermore, transfer learning models are also commonly utilized for malaria cell classification such as VGG16, AlexNet, DenseNet121, ResNet50, and Xception with an accuracy of 91.50–95.9%. In these transfer learning models, VGG16 and ResNet50 provided competent results compared to the others. Custom CNN models provided improved results compared to transfer learning and they gives an accuracy of 97.37% (20). The Dense Attentive Circular Network is used for malaria cell detection and achieved results are compared to the transfer learning models such as DPN92 and DenseNet121.This method provided an accuracy of 97.47 and 87.88% on DenseNet121 and DPN-92, respectively (21). The pre-trained networks such as ResNet50, AlexNet, and VGG19 are used for malaria classification and provide an accuracy of 93.88, 96.33, and 93.72%, respectively. Features are extracted from pre-trained VGG-16 and input to SVM for discrimination between infected/uninfected cells of malaria with 93.1% accuracy (22). Custom CNN (23–31) and pre-trained efficientnet-b0 model are used for features extraction and they provided accuracy of 97.74 and 98.82%, respectively (20). DCNN model is used for the classification of blood smear images with a 94.79% classification accuracy (32). The original malaria cell images are preprocessed based on the L^*^a^*^b^*^ and then extracted for deep features through Dense-Net-169 and Dense-Net 53 for deep feature analysis. Furthermore, the best features are selected using the whale optimization method for malaria cell classification with 99.67% accuracy (33). Malaria cells are classified using deep-sweep software with >0.95 ROC (34). Features are extracted from transfer learning models which are dense-net-201, dense-net-121, Resnet-101, Resnet-50, VGG-16, and VGG-19 for features extraction and input to SVM, NB, and KNN classifiers for malaria cell classification (35). LeNet, GoogLeNet, and AlexNet models are used for feature extraction with 94% accuracy for malaria cell classification (36). Mask-RCNN is used for malaria cell segmentation with a 94.57 correct rate (37). The faster-RCNN is used for model training with a single multi-shot detector SSD for localization of the malaria cells with a 0.94 prediction rate (38).

## Proposed methodology

**Figure 1:**
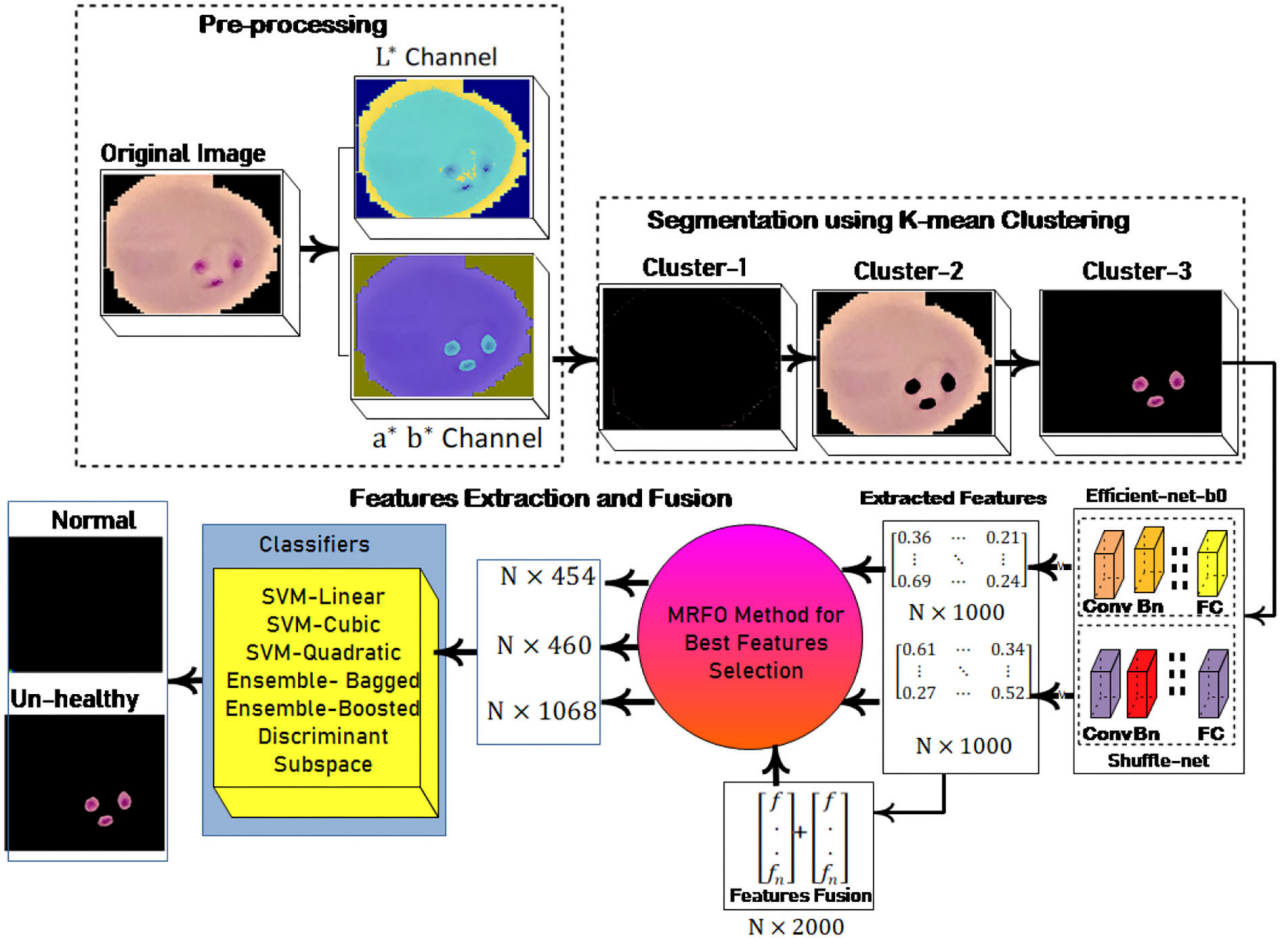
Proposed architecture for malaria classification.

In this research, a method is proposed for the segmentation and classification of malaria cells. The three-dimensional segmentation of the malaria cells is performed using K-mean clustering. After segmentation, the segmented images are fed to the proposed classification model. In this model, features are extracted from pre-trained models such as efficient-net-b0 and shuffle-net (39) with a dimension of *N* × 1,000. In which best *N* × 454 features from the shuffle-net model and *N* × 460 features from efficient-net-b0 (40) model. Furthermore, extracted features from both models are fused serially to create a fused feature vector (FV) with a length of *N* × 2,000. The best *N* × 1,068 features are selected out of *N* × 2,000 using the proposed MRFO method and passed to benchmark classifiers to classify the infected and un-infected malaria cells. The proposed method steps are illustrated in Figure 1, a normal image is black because it has no infected region.

### Segmentation of the malaria cells using K-mean clustering

The RGB input images φ are converted into the L^*^a^*^b^*^ color space for contrast adjustment. The mathematical representation is defined as:


(1) $$\begin{array}{l} \begin{array}{ccc} \varphi L^{{}^{*}} & = & 116\;h(\frac{Y}{Y_{\partial }})-16 \end{array}\\ \begin{array}{ccc} \varphi a^{{}^{*}} & = & 500\;[h(\frac{X}{X_{\partial }})-h(\frac{Y}{Y_{\partial }})] \end{array}\\ \begin{array}{ccc} \varphi b^{{}^{*}} & = & 200\;[h(\frac{Y}{Y_{\partial }})-h(\frac{Z}{Z_{\partial }}\;)] \end{array} \end{array}$$

Here *X*_∂_, *Y*_∂_, *Z*_∂_ denotes the white-tristimulus


$$h\left(f\right)=\left\{ \begin{array}{l} \sqrt[{3}]{\text{f}}\;f>0.008\\ 7.7f+\frac{16}{116}\;f\leq 0.008 \end{array}\right.$$

The K-mean clustering is applied to the luminance channel for segmentation. In K-mean clustering, we uniformly selected the observation centroid *c*_1_ from the input data X (41). The distance is computed to the observation to the *c*_1_ that is represented *d*(*x*_*m*_, *cj*). Then centroid c2 is selected randomly from the X data point with the probability is defined as follows:


(2) $$\begin{array}{r} \frac{d^{2}\left(x_{m},\;c1\right)\;}{\overset{n}{\underset{j=1}{\sum }}d^{2}\left(x_{j},\;c1\right)} \end{array}$$

**Figure 2:**
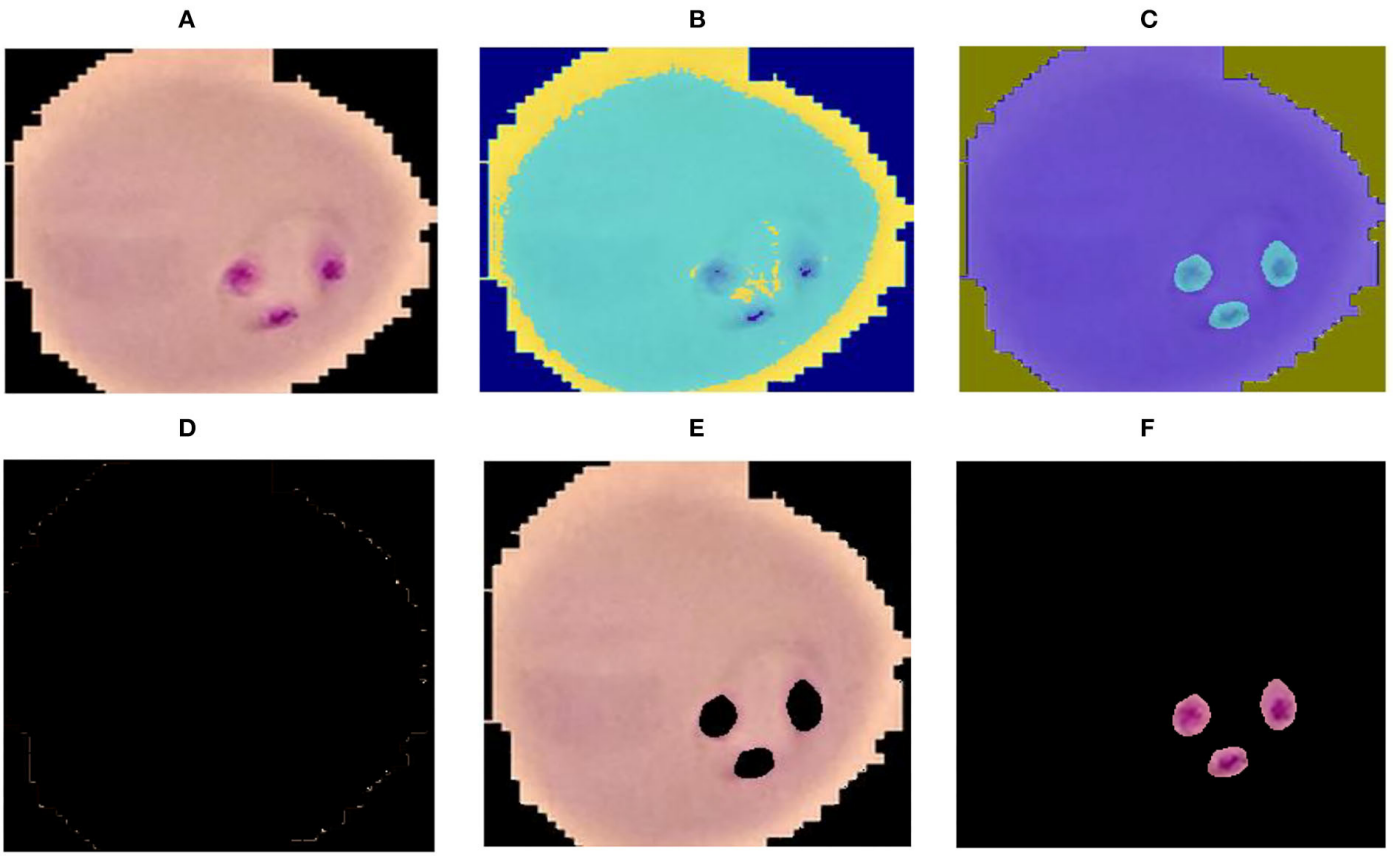
Segmentation results **(A)** input image **(B)** luminance channel **(C)** a and b channel **(D)** cluster-1 **(E)** cluster-2 and **(F)** cluster-3.

The distance from each observation to the centroid is computed and allocated observation to the nearest centroid is mathematically expressed as:


(3) $$\begin{array}{c} \frac{d^{2}(x_{m},\;c1)\;}{\sum_{\left\{h;x_{h\;\varepsilon cp}\right\}}d^{2}(x_{m},\;c1)} \end{array}$$

where *cp* denotes the closet centroid across each observation and *x*_*m*_ relate to the *cp*. In the proposed method malaria-infected region is segmented on the selected *k* = 3 values. The proposed segmentation results are shown in Figure 2.

### Malaria cells classification

**Figure 3:**
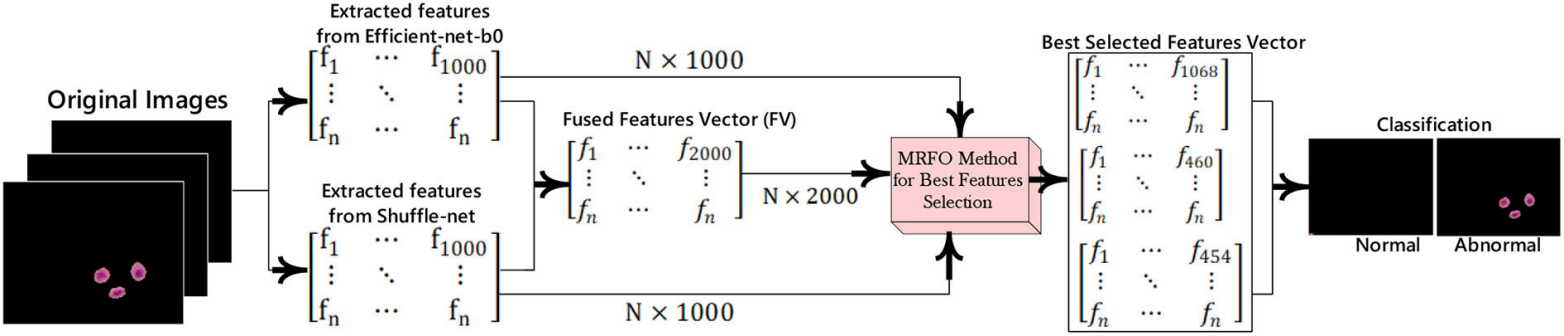
Best features extraction and selection process for malaria cell classification.

The malaria cells are classified using the proposed convolutional neural network-based transfer learning model such as efficientnet-b0 and shuffle-net. The pre-trained efficientnet-b0 consists of 290 layers such as convolutional (65), batch-normalization (49), sigmoid (65), element-wise multiplication (65), convolution group (15), average global pooling (16), addition (9), fully connected (FC), addition (15), classification, softmax, and global average pool (16). The shuffle-net consists of 172 layers such as input (1), convolution (49), (49) batch-normalization, ReLU (33), max-pooling (01), average pooling (02), 16 shuffling channels, 1 fully connected (FC), 1 softmax, 1 classification, 15 addition, 01 average global pool, and 2 depth concatenation. This research extracted features from the MatMul FC layer of efficient-netb0 and node-202 FC layer of shuffle-net. The extracted features are fused serially and fed to the MRFO method for the selection of optimum features. The proposed feature extraction and selection process are presented in Figure 3.

#### Best features selection using MRFO method

Manta Ray Foraging Optimization (MRFO) method is used for best feature selection. MRFO consists of three main steps such as chain fore-aging, cyclone fore-aging, and somersault fore-aging (42–44).

#### Chain fore-aging

Manta rays might see the direction of the plankton in MRFO and move toward it. The better the position is, the greater will be the concentration of plankton. Although the optimal answer is unknown, MRFO believes that the optimal solution discovered so far has been the plankton that comprises a greater concentration of manta rays that desire to reach and consume it. Manta rays construct a foraging chain by queuing up head to toe. Everyone else moves toward the meal and the individuals in front of it, except for first. So that, everyone gets updated by optimal answers found thus far and explanations ahead of it in each iteration. The mathematical representation of the chain foraging is defined as:


(4) $$\begin{array}{l} \;x_{i}{}^{d}\left(\omega +1\right)\\ \;=\{\begin{array}{c} x_{i}{}^{d}\left(\omega \right)+\partial .\left(x^{d}{}_{Best}\left(\omega \right)-x_{i}{}^{d}\left(\omega \right)\right)+\alpha .(x^{d}{}_{Best}\left(\omega \right)-x_{i}{}^{d}\left(\omega \right))\;i=1\\ x_{i}{}^{d}\left(\omega \right)+\partial .\left(x^{d}{}_{i-1}\left(\omega \right)-\;x_{i}{}^{d}\left(\omega \right)\right)+\alpha .\left(x^{d}{}_{Best}\left(\omega \right)-x_{i}{}^{d}\left(\omega \right)\right)\;i=2,\ldots N \end{array}\;\\ \alpha =2.\partial .\sqrt{\left|log\;(\partial )\right|} \end{array}$$

Here ∂ is a random value in the range of [ 0 1], α represents the learning rate, *d* denotes the dimension and ${x_{i}}^{d}\left(\omega \right)$ is the i individual at t time.

#### Cyclone foraging

When a group of the manta rays spots plankton in the intense water, they form an extensive foraging chain and travel in a spiral toward the meal. WOA uses a spiral foraging method that is comparable to this. According to the foraging technique of manta ray swarms, every manta ray moves toward the one ahead of it as well as spiraling toward the meal. The best answer identified through the cyclone foraging technique has better exploitation of the area because all individuals do the search at random with the food to their reference place. This trend is also employed to significantly enhance research. By allocating an unplanned place in the whole research area to the source point, we may need every participant to seek a different place.


(5) $$\begin{array}{l} \;x_{i}{}^{d}\left(\omega +1\right)\\ =\{\begin{array}{c} x_{i}{}^{d}\left(\omega \right)+\partial .\left(x^{d}{}_{Best}\left(\omega \right)-x_{i}{}^{d}\left(\omega \right)\right)+\beta .(x^{d}{}_{Best}\left(\omega \right)-x_{i}{}^{d}\left(\omega \right))\;i=1\\ x_{i}{}^{d}\left(\omega \right)+\partial .\left(x^{d}{}_{i-1}\left(\omega \right)-\;x_{i}{}^{d}\left(\omega \right)\right)+\beta .\left(x^{d}{}_{Best}\left(\omega \right)-x_{i}{}^{d}\left(\omega \right)\right)\;i=2,\ldots N \end{array} \end{array}$$

$\beta =2e^{\partial_{1\;}\frac{T-t+1}{T}}.\;\sin\left(2\pi r_{1}\right)$ where β represents the coefficient of weights and *T* is the total iterations.

#### Somersault fore-aging

This technique is mainly involved with research and permits MRFO to organize global research. The meal position is considered a hinge in these behaviors. Every person moves to and fro around the hinge, somersaulting to the given assignment. Consequently, they always adjust their placements to the greatest possible position found until now.


(6) $$\begin{array}{l} x_{i}{}^{d}\left(\omega +1\right)=x_{i}{}^{d}\left(\omega \right)+S\;.\;\left(\partial_{2}\;.\;x^{d}{}_{Best}-\partial_{3}\;.\;x_{i}{}^{d}\left(\omega \right)\right),\\ \;i=1\ldots ..N \end{array}$$

Here's denote the range of the somersault factor, *S* = 2, ∂_2_, ∂_3_ are random two numbers in [ 0.1] range. In this research parameters of MRFO are selected for best features selection as presented in Table 1.

**Table 1:** Selection of the best features using MRFO.

**Lower bound**	**Upper bound**	**Threshold**	**Somersault**	**Total iterations**	**Total solutions**	**Classification error rate**
***0***	***1***	***0.5***	***2***	***100***	***10***	***0.008***
		0.6	3		12	0.009
		0.4	1		09	0.020
		0.7	4		11	0.031
		0.3	5		08	0.091

**Figure 4:**
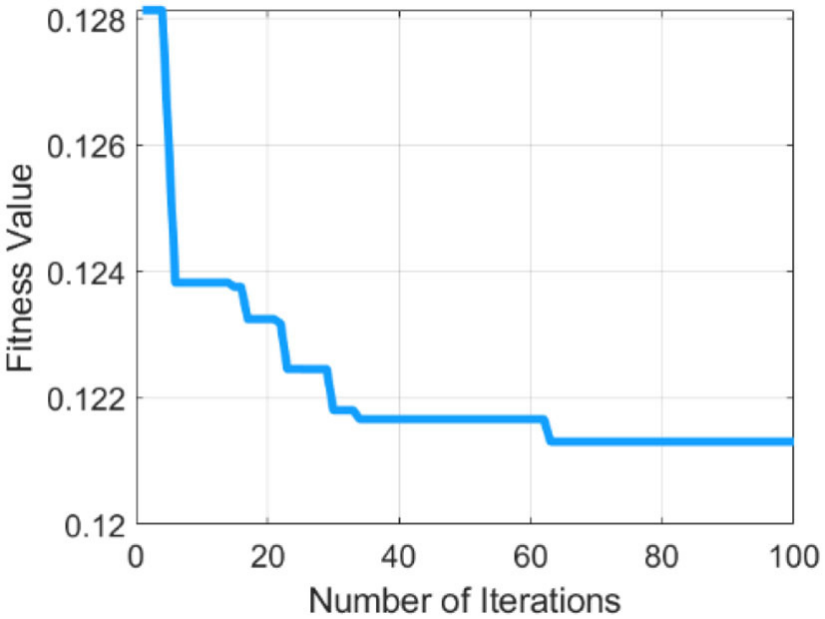
Graphical representation of the MRFO method.

Table 1 describes the parameters of MRFO, in which 0.5 threshold,two Somersault, and 100 iterations with 10 total solutions are used for model training due to less error rate these are selected for further processing. This experimental model is converge after the 60 epochs on a 0.128 fitness value as presented in Figure 4.

This method is applied to individual feature vector and the fusion of both feature vectors. In this experiment, we achieve the dimension of *N* × 454 features from shuffle net and *N* × 460 features from efficient-net-b0 features. Finally, achieved *N*× 1,068 best-selected features after the fusion of both feature vectors. The selected feature vectors are fed to the ensemble and SVM classifiers for malaria cell classification.

## Results and discussion

The proposed method results are evaluated on a publically available malaria benchmark dataset. This dataset contains 2,750 of two classes in which 1,375 infected and 1,375 un-infected images are included (45). The proposed method of experimentation is implemented on the Core-i7 window operating system, Nvidia 2070-RTX GPU.

### Experiment#1: Malaria classification

The input microscopic malaria cells are segmented using color-based k-mean clustering. In this experiment malaria, cells are classified into two classes using precision (Pi), accuracy (Accy), recall (Rec), and F1-score (F1e) as presented in Tables 2, 3. The proposed method classification results using efficient-net-b0 are shown in Table 2.

**Table 2:** Classification of malaria cells using efficient-net-b0 features.

**Classifier**	**Infected**	**Un-infected**	**Accy**	**Pi**	**Rec**	**F1e**
Linear	✓		94.68%	0.95	0.95	0.95
		✓	94.68%	0.95	0.95	0.95
Quadratic	✓		93.87%	0.93	0.94	0.94
		✓	93.87%	0.94	0.93	0.94
Cubic	✓		92.22%	0.92	0.92	0.92
		✓	92.22%	0.92	0.92	0.92
Boosted	✓		91.52%	0.92	0.92	0.92
		✓	91.52%	0.90	0.91	0.91
Bagged	✓		91.31%	0.93	0.92	0.92
		✓	91.31%	0.90	0.91	0.90
Subspace discriminant	✓		92.62%	0.95	0.94	0.94
		✓	92.62%	0.90	0.91	0.90

**Table 3:** ROC-AUC values on benchmark classifiers using efficient-net-b0.

**Boosted**	**Bagged**	**Subspace discriminant + boosted**	**Subspace discriminant + bagged**	**Linear**	**Cubic**	**Quadratic + linear**	**Quadratic + cubic**	**ROC-AUC**
✓								0.737
	✓							0.729
		✓						0.726
			✓					0.727
				✓				0.738
					✓			0.726
						✓		0.758
							✓	0.760

In Table 2, features are extracted from the efficientnet-b0 and the best features are selected using MRFO that are fed to the SVM and ensemble classifiers. In this experiment, we achieved a maximum accuracy of 94.68% using a linear classifier as compared to others. Ten-fold cross-validation is used to calculate the categorization results using the best-selected features vector dimension of *N* × 454 from the shuffle-net as presented in Table 3.

**Figure 5:**
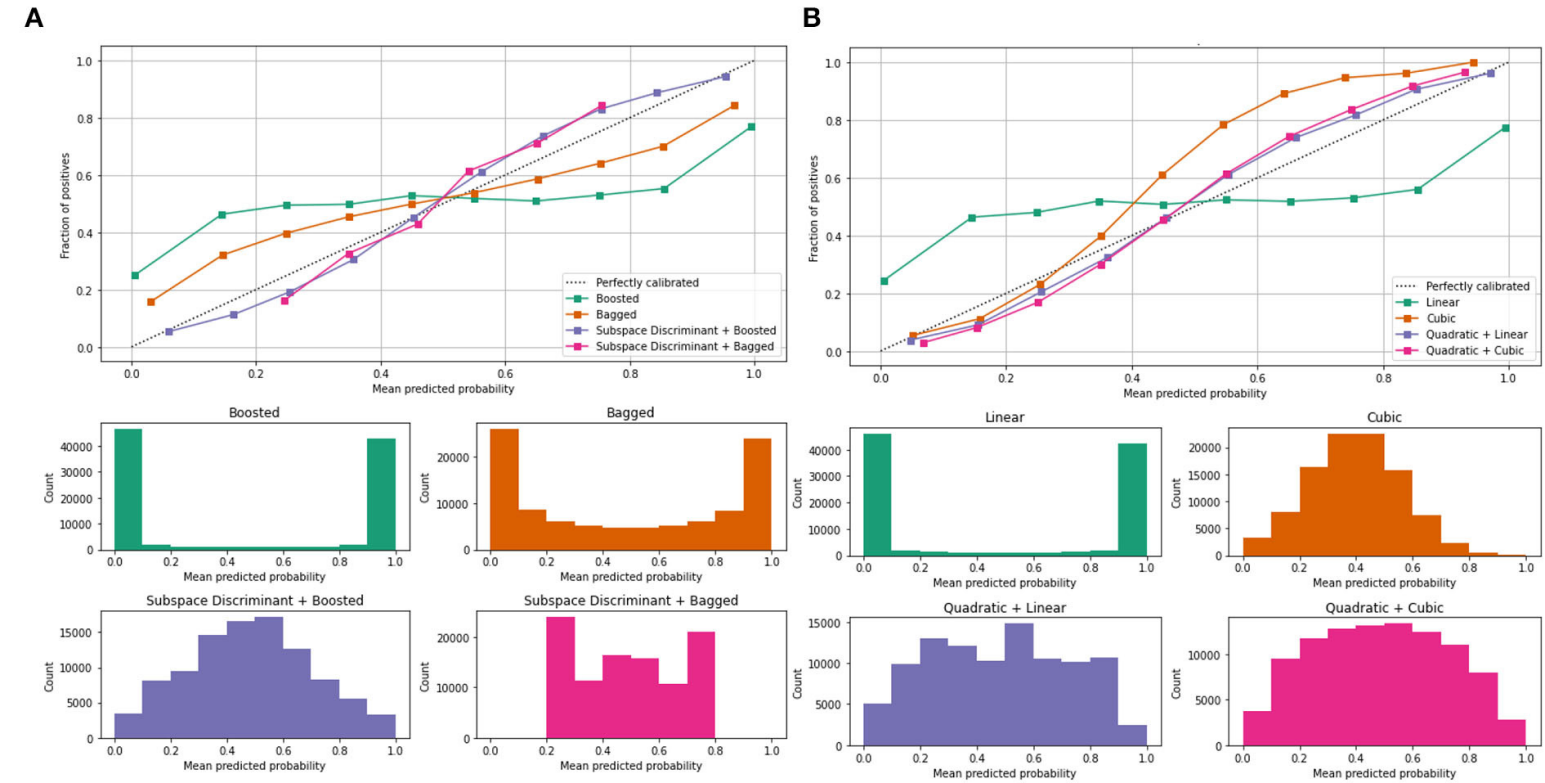
Mean predicted scores on the benchmark classifiers **(A)** ensemble **(B)** SVM. The ROC-AUC values are computed on benchmark classifiers as presented in Table 3.

Mean predicted scores are also computed to authenticate the proposed method performance as shown in Table 4; Figure 5.

**Table 4:** Classification results using shuffle-net.

**Classifier**	**Infected**	**Un-infected**	**Accy**	**Pi**	**Rec**	**F1e**
Linear	✓		93.67%	0.95	0.93	0.94
		✓	93.67%	0.92	0.94	0.93
Quadratic	✓		94.87%	0.96	0.95	0.96
		✓	94.87%	0.93	0.95	0.94
Cubic	✓		95.16%	0.96	0.95	0.96
		✓	95.16%	0.94	0.95	0.94
Boosted	✓		93.68%	0.94	0.95	0.95
		✓	93.68%	0.93	0.92	0.93
Bagged	✓		94.63%	0.95	0.96	0.95
		✓	94.63%	0.94	0.93	0.93
Subspace discriminant	✓		94.76%	0.94	0.97	0.96
		✓	94.76%	0.95	0.92	0.93

In Table 3, a significant test is performed in which the ROC–AUC value is computed on bagged, boosted, linear, cubic classifiers, and fusion of subspace discriminant + boosted, subspace discriminant + bagged, quadratic + linear, quadratic + cubic classifiers. This experiment achieved the highest ROC-AUC of 0.760 using the fusion of quadratic + cubic classifiers. The classification results based on shuffle-net features are presented in Table 4.

**Figure 6:**
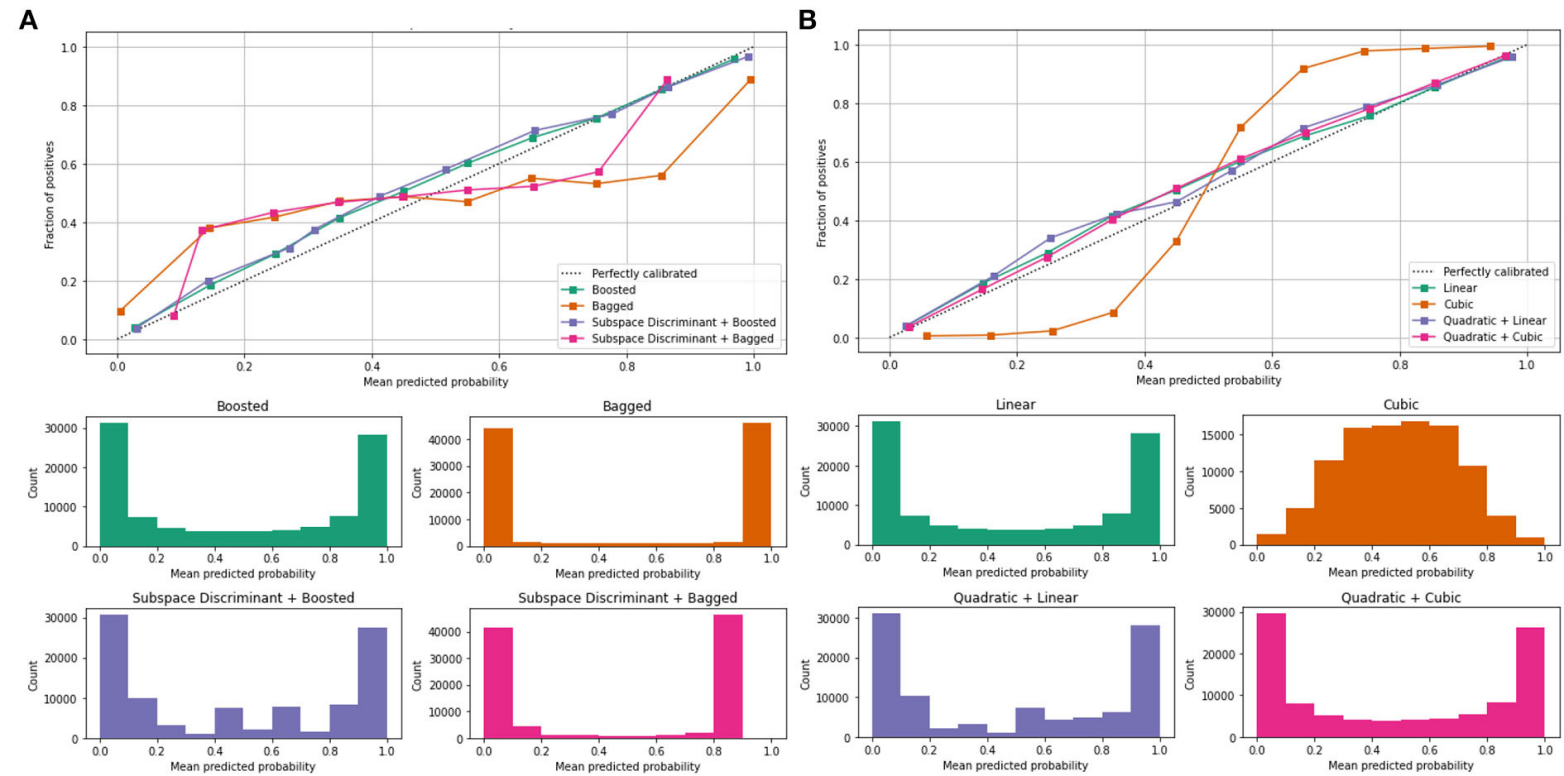
Mean predicted scores using shuffle-net features vector **(A)** ensemble and **(B)** SVM.

The classification results are mentioned in Table 4, in which we achieved 95.16 accuracies on the SVM cubic classifier, which is far better compared to others. Furthermore, classification results are also computed in terms of ROC–AUC, in which classifiers are used individually as well as the fusion of the boosted, bagged, linear, and cubic kernels with the subspace discriminant and quadratic, respectively. The quantitative analysis of the features vector obtained from the shuffle net is presented in Figure 6; Table 5.

**Table 5:** Quantitative results for malaria classification using shuffle-net features.

**Boosted**	**Bagged**	**Subspace discriminant + boosted**	**Subspace discriminant + bagged**	**Linear**	**Cubic**	**Quadratic + linear**	**Quadratic + cubic**	**ROC-AUC**
✓								0.863
	✓							0.865
		✓						0.862
			✓					0.865
				✓				0.863
					✓			0.863
						✓		0.863
							✓	0.862

Figure 6 shows the mean predicted scores on the benchmark classifiers. The quantitative computed results are mentioned in Table 5.

In the classification of malaria cells, we achieved a ROC–AUC of 0.865 using a bagged and subspace discriminant + bagged classifier. The classification results are computed using the fused features vector as presented in Table 6.

**Table 6:** Classification of malaria cells using fused features vector.

**Classifier**	**Infected**	**Un-infected**	**Accy**	**Pi**	**Rec**	**F1e**
Linear	✓		99.2%	0.99	0.99	0.99
		✓	99.2%	0.99	0.99	0.99
Quadratic	✓		97.64%	0.98	0.97	0.98
		✓	97.64%	0.97	0.98	0.98
Cubic	✓		95.97%	0.97	0.95	0.96
		✓	95.97%	0.95	0.96	0.96
Boosted	✓		96.17%	0.97	0.96	0.96
		✓	96.17%	0.96	0.97	0.96
Bagged	✓		96.39%	0.97	0.96	0.96
		✓	96.39%	0.96	0.97	0.96
Subspace discriminant	✓		97.49%	1.00	0.95	0.97
		✓	97.49%	0.96	1.00	0.98

In the results in Table 6, after applying the fused features vector, we obtained an accuracy of 99.2% on the linear classifier and 97.64% on the quadratic kernel. The fused features vector provides excellent results compared to the individual features vector that authenticates the proposed method novelty. The mean of the correct prediction scores on the fused features vector is presented in Table 7.

**Table 7:** Classification of malaria cells in terms of mean predicted scores using fused features vector.

**Boosted**	**Bagged**	**Subspace discriminant + boosted**	**Subspace discriminant + bagged**	**Linear**	**Cubic**	**Quadratic + linear**	**Quadratic + cubic**	**ROC-AUC**
✓								0.951
	✓							0.952
		✓						0.951
			✓					0.952
				✓				0.951
					✓			0.951
						✓		0.951
							✓	0.951

**Figure 7:**
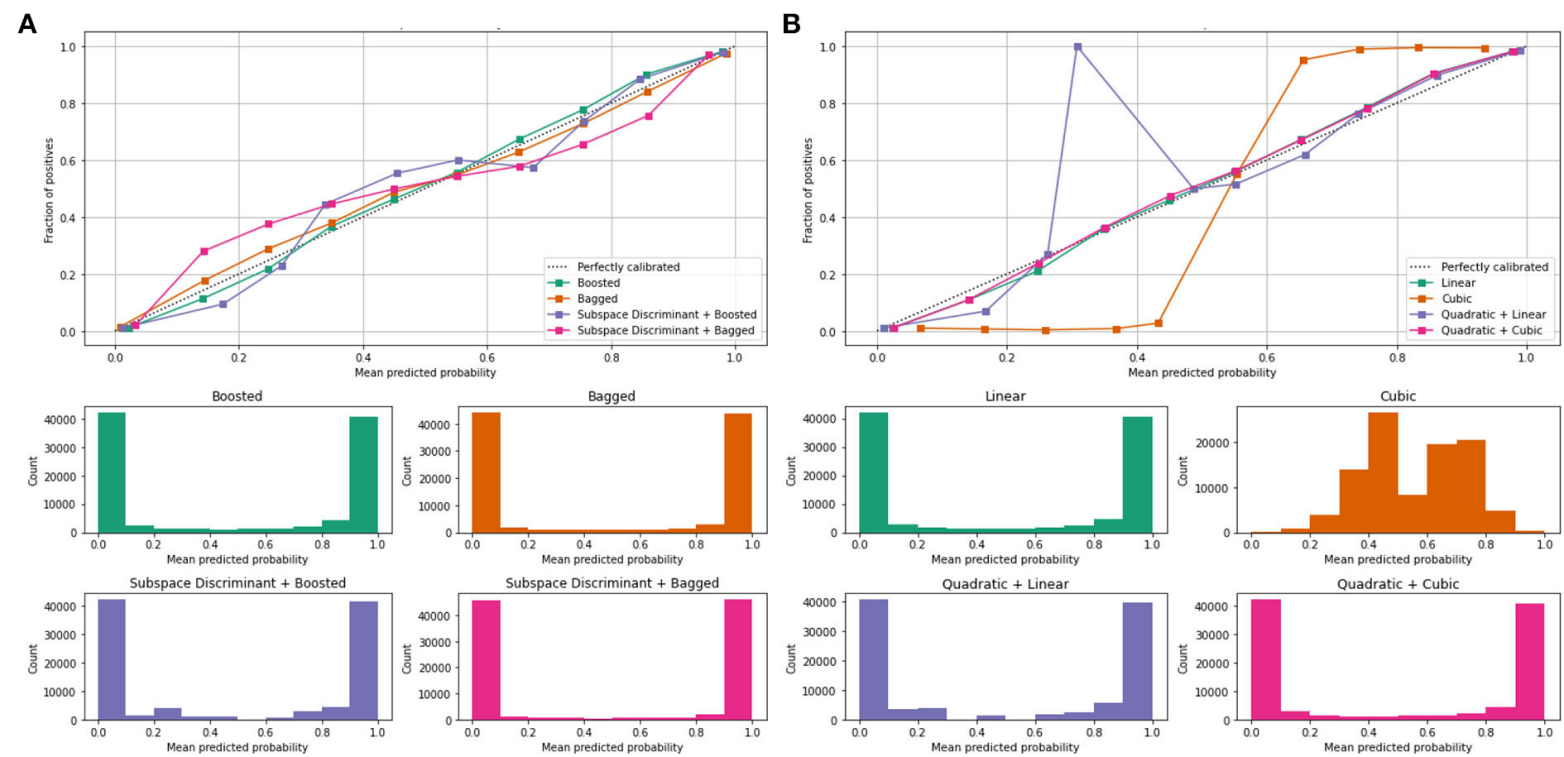
Mean predicted scores using fused features vector **(A)** boosted, bagged, and discriminant kernels of ensemble classifier and **(B)** linear, cubic, and quadratic kernels of SVM.

Mean values of the correct prediction scores are presented in Table 7, in which the highest ROC-AUC of 0.952 using bagged and subspace discriminant + bagged classifier as compared to others. In Figure 7, a combination of the ROC curve of the selected classifier is plotted as well as the mean predicted probability of the benchmark kernels such as linear, cubic, quadratic, boosted, bagged, and discriminant is plotted.

Figure 7 shows the computed classification results of the individual and fusion of the bags with discriminate, linear with quadratic, and quadratic with cubic. The proposed method results are compared to the current existing methods as presented in Table 8.

**Table 8:** Proposed classification method results comparison.

**Ref#**	**Year**	**Accuracy**
(46)	2022	97.68%
(47)	2022	94.00% using SVM, 90.00% XG-Boost, 80.00% and neural networks classifier
(32)	2022	94.79%
(48)	2022	94.17%
(49)	2022	96.00%
**Proposed Method**		99.20%

In Table 8, the proposed classification method results are contrasted with the most recent works published on the same benchmark dataset. In which the pre-trained ResNet-18 model is used for malaria classification it gives an accuracy of 98.68% (46). The classifiers SVM, neural network, and XG-boost are used for malaria classification with the accuracy of 94, 90, and 80%, respectively (47). The DAG–CNN model is used for parasite malaria classification and it gives an accuracy of 94.79% (32). NC–GCN model is used for feature extraction and malaria cells are classified with 94.17% accuracy (48). The pre-trained inception-v3 and VGG-19 models are used for feature extraction. The average achieved accuracy is 96%.

Compared to the most recent techniques used in this research a method is proposed to investigate the deep features analysis using efficient-net-b0 and shuffle-net. These features are used for classification in two ways. First, the MRFO method is applied to the extracted features of both models and the best-selected features are passed to the classifiers. In the second way, extracted features are serially fused and optimum features are selected using the MRFO method. In this experiment, we observed that the fused features vector performs significant improvement in malaria cell classification.

## Conclusion

Parasite malaria segmentation and classification is an intricate task due to the large variation and illumination in microscopic malaria images. Therefore, in this research, a method is proposed in which the quality of the input images is improved by applying a pre-processing method. The RGB microscopic malaria images are converted into CIELab color space. The luminance channel is selected for further processing to improve the image contrast. The best-segmented results of the malaria cells are achieved in the third cluster. The segmented images are fed to the proposed classification model. The proposed method more accurately classifies the malaria cells due to features fusion, and optimum features selected by the MRFO method. The proposed method provides an accuracy of 95.16% on the individual best-selected features vector and 99.2% on the fused best-selected features vector. Furthermore, to authenticate the performance of the proposed classification method ROC-AUC values are computed using individual classifiers such as linear, cubic, bagged, boosted, and the fusion of cubic + quadratic, and linear + quadratic, bagged + subspace discrimination, boosted + subspace + discriminant classifiers. In this experiment highest, the 0.95 ROC-AUC was achieved using a bagged + subspace discriminant classifier. In comparison to the most recent approaches, the experimental results show that the fused features vector produces the best outcomes.

## Data availability statement

The original contributions presented in the study are included in the article/supplementary files, further inquiries can be directed to the corresponding author/s.

## Author contributions

JA performed writing draft, conceptualization, and implementation. MS contributed as the part of result validation team and writing the conclusion of the paper. SF and GM contributed to the data curation, investigation, and literature reviews. SF contributed to the analysis and editing the original draft. GM contributed to the resources and project administration. All authors contributed to the article and approved the submitted version.

## Conflict of interest

The authors declare that the research was conducted in the absence of any commercial or financial relationships that could be construed as a potential conflict of interest.

## Publisher's note

All claims expressed in this article are solely those of the authors and do not necessarily represent those of their affiliated organizations, or those of the publisher, the editors and the reviewers. Any product that may be evaluated in this article, or claim that may be made by its manufacturer, is not guaranteed or endorsed by the publisher.
